# A hybrid and scalable error correction algorithm for indel and substitution errors of long reads

**DOI:** 10.1186/s12864-019-6286-9

**Published:** 2019-12-20

**Authors:** Arghya Kusum Das, Sayan Goswami, Kisung Lee, Seung-Jong Park

**Affiliations:** 1 Department of Computer Science and Software Engineering, University of Wisconsin at Platteville, Platteville, WI USA; 2 School of Electrical Engineering and Computer Science, Center for Computation and Technology, Louisiana State University, Baton Rouge, Baton Rouge, LA USA

**Keywords:** Hybrid error correction, PacBio, Illumina, Hadoop, NoSQL

## Abstract

**Background:**

Long-read sequencing has shown the promises to overcome the short length limitations of second-generation sequencing by providing more complete assembly. However, the computation of the long sequencing reads is challenged by their higher error rates (e.g., 13% vs. 1%) and higher cost ($0.3 vs. $0.03 per Mbp) compared to the short reads.

**Methods:**

In this paper, we present a new hybrid error correction tool, called ParLECH (Parallel Long-read Error Correction using Hybrid methodology). The error correction algorithm of ParLECH is distributed in nature and efficiently utilizes the *k*-mer coverage information of high throughput Illumina short-read sequences to rectify the PacBio long-read sequences.ParLECH first constructs a de Bruijn graph from the short reads, and then replaces the indel error regions of the long reads with their corresponding widest path (or maximum min-coverage path) in the short read-based de Bruijn graph. ParLECH then utilizes the *k*-mer coverage information of the short reads to divide each long read into a sequence of low and high coverage regions, followed by a majority voting to rectify each substituted error base.

**Results:**

ParLECH outperforms latest state-of-the-art hybrid error correction methods on real PacBio datasets. Our experimental evaluation results demonstrate that ParLECH can correct large-scale real-world datasets in an accurate and scalable manner. ParLECH can correct the indel errors of human genome PacBio long reads (312 GB) with Illumina short reads (452 GB) in less than 29 h using 128 compute nodes. ParLECH can align more than 92% bases of an *E. coli* PacBio dataset with the reference genome, proving its accuracy.

**Conclusion:**

ParLECH can scale to over terabytes of sequencing data using hundreds of computing nodes. The proposed hybrid error correction methodology is novel and rectifies both indel and substitution errors present in the original long reads or newly introduced by the short reads.

## Background

The rapid development of genome sequencing technologies has become the major driving force for genomic discoveries. The second-generation sequencing technologies (e.g., Illumina, Ion Torrent) have been providing researchers with the required throughput at significantly low cost ($0.03/million-bases), which enabled the discovery of many new species and variants. Although they are being widely utilized for understanding the complex phenotypes, they are typically incapable of resolving long repetitive elements, common in various genomes (e.g., eukaryotic genomes), because of the short read lengths [1].

To address the issues with the short read lengths, third-generation sequencing technologies (e.g., PacBio, Oxford Nanopore) have started emerging recently. By producing long reads greater than 10 kbp, these third-generation sequencing platforms provide researchers with significantly less fragmented assembly and the promise of a much better downstream analysis. However, the production costs of these long sequences are almost 10 times more expensive than those of the short reads, and the analysis of these long reads is severely constrained by their higher error rate.

Motivated by this, we develop ParLECH (Parallel Long-read Error Correction using Hybrid methodology). ParLECH uses the power of MapReduce and distributed NoSQL to scale with terabytes of sequencing data [2]. Utilizing the power of these big data programming models, we develop fully distributed algorithms to replace both the indel and substitution errors of long reads. To rectify the indel errors, we first create a de Bruijn graph from the Illumina short reads. The indel errors of the long reads are then replaced with the widest path algorithm that maximizes the minimum *k*-mer coverage between two vertices in the de Bruijn graph. To correct the substitution errors, we divide the long read into a series of low and high coverage regions by utilizing the median statistics of the *k*-mer coverage information of the Illumina short reads. The substituted error bases are then replaced separately in those low and high coverage regions.

ParLECH can achieve higher accuracy and scalability over existing error correction tools. For example, ParLECH successfully aligns 95% of *E. Coli* long reads, maintaining larger N50 compared to the existing tools. We demonstrate the scalability of ParLECH by correcting a 312GB human genome PacBio dataset, with leveraging a 452 GB Illumina dataset (64x coverage), on 128 nodes in less than 29 h.

### Related work

The second-generation sequencing platforms produce short reads at an error rate of 1-2% [3] in which most of the errors are substitution errors. However, the low cost of production results in high coverage of data, which enables self-correction of the errors without using any reference genome. Utilizing the basic fact that the *k*-mers resulting from an error base will have significantly lower coverage compared to the actual *k*-mers, many error correction tools have been proposed such as Quake [4], Reptile [5], Hammer [6], RACER [7], Coral [8], Lighter [9], Musket [10], Shrec [11], DecGPU [12], Echo [13], and ParSECH [14].

Unlike second-generation sequencing platforms, the third-generation sequencing platforms, such as PacBio and Oxford Nanopore sequencers, produce long reads where indel (insertion/deletion) errors are dominant [1]. Therefore, the error correction tools designed for substitution errors in short reads cannot produce accurate results for long reads. However, it is common to leverage the relatively lower error rate of the short-read sequences to improve the quality of long reads.

While improving the quality of long reads, these hybrid error correction tools also reduce the cost of the pipeline by utilizing the complementary low-cost and high-quality short reads. LoRDEC [15], Jabba [16], Proovread [17], PacBioToCA [18], LSC [19], and ColorMap [20] are a few examples of hybrid error correction tools. LoRDEC [15] and Jabba [16] use a de Bruijn graph (DBG)-based methodology for error correction. Both the tools build the DBG from Illumina short reads. LoRDEC then corrects the error regions in long reads through the local assembly on the DBG while Jabba uses different sizes of *k*-mer iteratively to polish the unaligned regions of the long reads. Some hybrid error correction tools use alignment-based approaches for correcting the long reads. For example, PacBioToCA [18] and LSC [19] first map the short reads to the long reads to create an overlap graph. The long reads are then corrected through a consensus-based algorithm. Proovread [17] reaches the consensus through the iterative alignment procedures that increase the sensitivity of the long reads incrementally in each iteration. ColorMap [20] keeps information of consensual dissimilarity on each edge of the overlap graph and then utilizes the Dijkstra’s shortest path algorithm to rectify the indel errors. Although these tools produce accurate results in terms of successful alignments, their error correction process is lossy in nature, which reduces the coverage of the resultant data set. For example, Jabba, PacBioToCA, and Proovread use aggressive trimming of the error regions of the long reads instead of correcting them, losing a huge number of bases after the correction [21] and thereby limiting the practical use of the resultant data sets. Furthermore, these tools use a stand-alone methodology to improve the base quality of the long reads, which suffers from scalability issues that limit their practical adoption for large-scale genomes.

On the contrary, ParLECH is distributed in nature, and it can scale to terabytes of sequencing data on hundreds of compute nodes. ParLECH utilizes the DBG for error correction like LoRDEC. However, to improve the error correction accuracy, we propose a widest path algorithm that maximizes the minimum *k*-mer coverage between two vertices of the DBG. By utilizing the *k*-mer coverage information during the local assembly on the DBG, ParLECH is capable to produce more accurate results than LoRDEC. Unlike Jabba, PacBioToCA, and Proovread, ParLECH does not use aggressive trimming to avoid lossy correction. ParLECH further improves the base quality instead by correcting the substitution errors either present in the original long reads or newly introduced by the short reads during the hybrid correction of the indel errors. Although there are several tools to rectify substitution errors for second-generation sequences (e.g., [4, 5, 9, 13]), this phase is often overlooked in the error correction tools developed for long reads. However, this phase is important for hybrid error correction because a significant number of substitution errors are introduced by the Illumina reads. Existing pipelines depend on polishing tools, such as Pilon [22] and Quiver [23], to further improve the quality of the corrected long reads. Unlike the distributed error correction pipeline of ParLECH, these polishing tools are stand-alone and cannot scale with large genomes.

LorMA [24], CONSENT [25], and Canu [26] are a few self-error correction tools that utilize long reads only to rectify the errors in them. These tools can automatically bypass the substitution errors of the short reads and are capable to produce accurate results. However, the sequencing cost per base for long reads is extremely high, and so it would be prohibitive to get long reads with high coverage that is essential for error correction without reference genomes. Although Canu reduces the coverage requirement to half of that of LorMA and CONSENT by using the tf-idf weighting scheme for long reads, almost 10 times more expensive cost of PacBio sequences is still a major obstacle to utilizing it for large genomes. Because of this practical limitation, we do not report the accuracy of the these self-error correction tools in this paper.

## Methods

### Rationale behind the indel error correction

Since we leverage the lower error rate of Illumina reads to correct the PacBio indel errors, let us first describe an error model for Illumina sequences and its consequence on the DBG constructed from these reads. We first observe that *k*-mers, DNA words of a fixed length *k*, tend to have similar abundances within a read. This is a well-known property of *k*-mers that stem from each read originating from a single source molecule of DNA [27]. Let us consider two reads *R*_1_ and *R*_2_ representing the same region of the genome, and *R*1 has one error base. Assuming that the *k*-mers between the position *pos*_*begin*_ and *pos*_*end*_ represent an error region in *R*_1_ where error base is at position ${pos}_{error} = \frac {pos_{end}+{pos}_{begin}}{2}$, we can make the following claim.

**Fig. 1:**
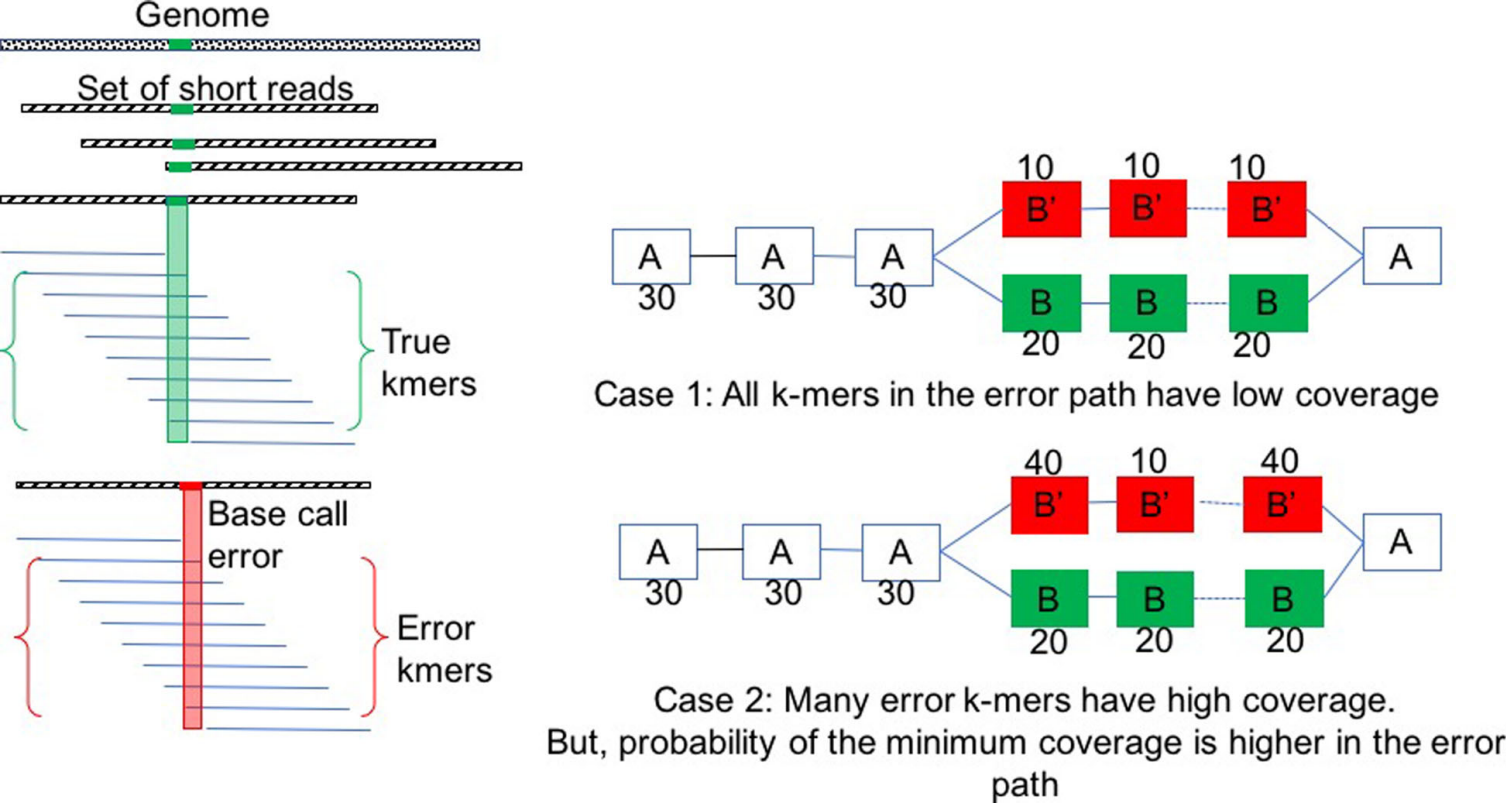
Widest Path Example: Select correct path for high coverage error *k*-mers

*Claim 1:* The coverage of at least one *k*-mer of *R*_1_ in the region between *pos*_*begin*_ and *pos*_*end*_ is lower than the coverage of any *k*-mer in the same region of *R*_2_. A brief theoretical rationale of the claim can be found in Additional file 1. Figure 1 shows the rationale behind the claim.

### Rationale behind the substitution error correction

After correcting the indel errors with the Illumina reads, a substantial number of substitution errors are introduced in the PacBio reads as they dominate in the Illumina short-read sequences. To rectify those errors, we first divide each PacBio long read into smaller subregions like short reads. Next, we classify only those subregions as errors where most of the *k*-mers have high coverage, and only a few low-coverage *k*-mers exist as outliers.

**Fig. 2:**
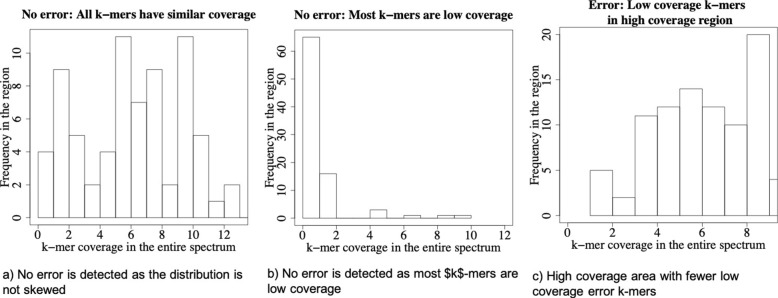
Skewness in *k*-mer coverage statistics

Specifically, we use Pearson’s skew coefficient (or median skew coefficient) to classify the true and error subregions. Figure 2 shows the histogram of three different types of subregions in a genomic dataset. Figure 2a has similar numbers of low- and high-coverage *k*-mers, making the skewness of this subregion almost zero. Hence, it is not considered as error. Figure 2b is also classified as true because the subregion is mostly populated with the low-coverage *k*-mers. Figure 2c is classified as error because the subregion is largely skewed towards the high-coverage *k*-mers, and only a few low-coverage *k*-mers exist as outliers. Existing substitution error correction tools do not analyze the coverage of neighboring *k*-mers and often classify the true yet low-coverage *k*-mers (e.g., Fig. 2b as errors.

Another major advantage of our median-based methodology is that the accuracy of the method has a lower dependency on the value of *k*. Median values are robust because, for a relatively small value of *k*, a few substitution errors will not alter the median *k*-mer abundance of the read [28]. However, these errors will increase the skewness of the read. The robustness of the median values in the presence of sequencing errors is shown mathematically in the Additional file 1.

### Big data framework in the context of genomic error correction

Error correction for sequencing data is not only data- and compute-intensive but also search-intensive because the size of the *k*-mer spectrum increases almost exponentially with the increasing value of *k* (i.e., up to 4^*k*^ unique *k*-mers), and we need to search in the huge search space. For example, a large genome with 1 million reads of length 5000 bp involves more than 5 billion searches in a set of almost 10 billion unique *k*-mers. Since existing hybrid error correction tools are not designed for large-scale genome sequence data such as human genomes, we design ParLECH as a scalable and distributed framework equipped with Hadoop and Hazelcast.

Hadoop is an open-source abstraction of Google’s MapReduce, which is a fully parallel and distributed framework for large-scale computation. It reads the data from a distributed file system called Hadoop Distributed File System (HDFS) in small subsets. In the Map phase, a Map function executes on each subset, producing the output in the form of key-value pairs. These intermediate key-value pairs are then grouped based on the unique keys. Finally, a Reduce function executes on each group, producing the final output on HDFS.

Hazelcast [29] is a NoSQL database, which stores large-scale data in the distributed memory using a key-value format. Hazelcast uses MummurHash to distribute the data evenly over multiple nodes and to reduce the collision. The data can be stored and retrieved from Hazelcast using hash table functions (such as *get* and *put*) in *O*(1) time. Multiple Map and Reduce functions can access this hash table simultaneously and independently, improving the search performance of ParLECH.

### Error correction pipeline

**Fig. 3:**
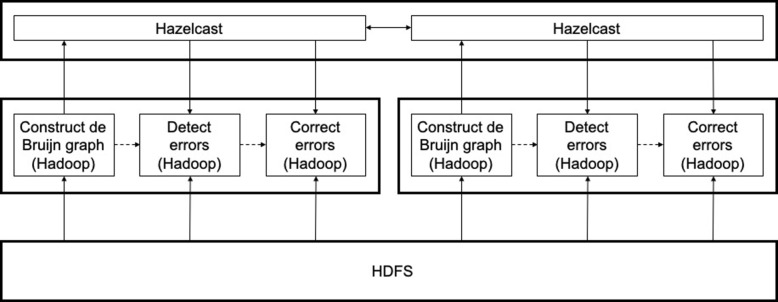
Indel error correction

Figure 3 shows the indel error correction pipeline of ParLECH. It consists of three phases: 1) constructing a de Bruijn graph, 2) locating errors in long reads, and 3) correcting the errors. We store the raw sequencing reads in the HDFS while Hazelcast is used to store the de Bruijn graph created from the Illumina short reads. We develop the graph construction algorithm following the MapReduce programming model and use Hadoop for this purpose. In the subsequent phases, we use both Hadoop and Hazelcast to locate and correct the indel errors. Finally, we write the indel error-corrected reads into HDFS. We describe each phase in detail in the subsequent sections.

**Fig. 4:**
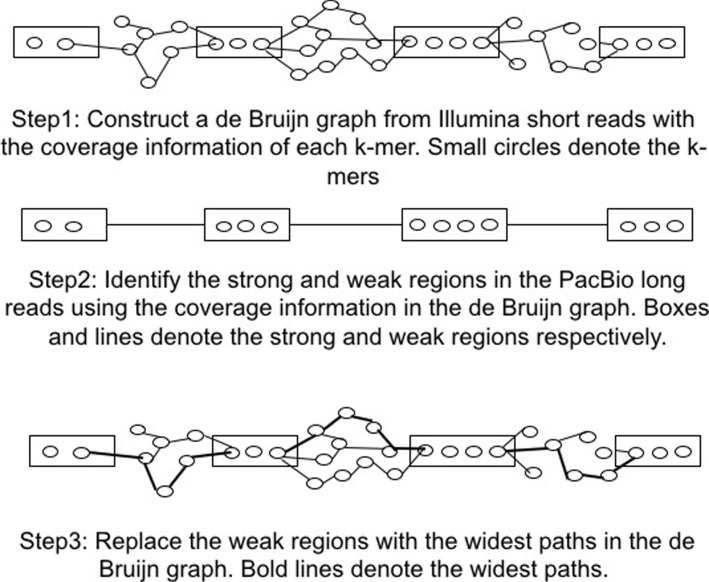
Error correction steps

ParLECH has three major steps for hybrid correction of indel errors as shown in Fig. 4. In the first step, we construct a DBG from the Illumina short reads with the coverage information of each *k*-mer stored in each vertex. In the second step, we partition each PacBio long read into a sequence of strong and weak regions (alternatively, correct and error regions respectively) based on the *k*-mer coverage information stored in the DBG. We select the right and left boundary *k*-mers of two consecutive strong regions as source and destination vertices respectively in the DBG. Finally, in the third step, we replace each weak region (i.e., indel error region) of the long read between those two boundary *k*-mers with the corresponding widest path in the DBG, which maximizes the minimum *k*-mer coverage between those two vertices.

**Fig. 5:**
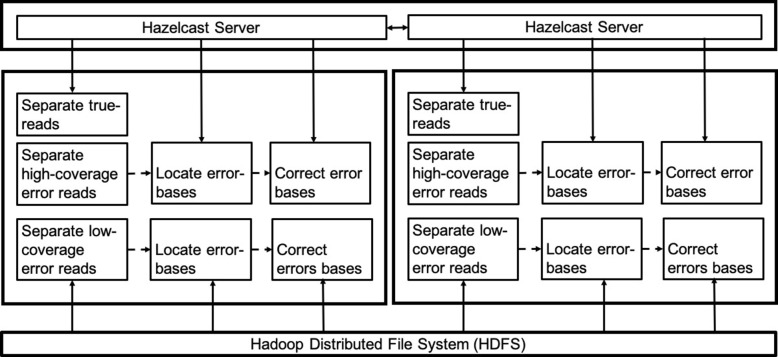
Substitution error correction

Figure 5 shows the substitution error correction pipeline of ParLECH. It has two different phases: 1) locating errors and 2) correcting errors. Like the indel error correction, the computation of phase is fully distributed with Hadoop. These Hadoop-based algorithms work on top of the indel error-corrected reads that were generated in the last phase and stored in HDFS. The same *k*-mer spectrum that was generated from the Illumina short reads and stored in Hazelcast is used to correct the substitution errors as well.

### De bruijn graph construction and counting *k*-mer


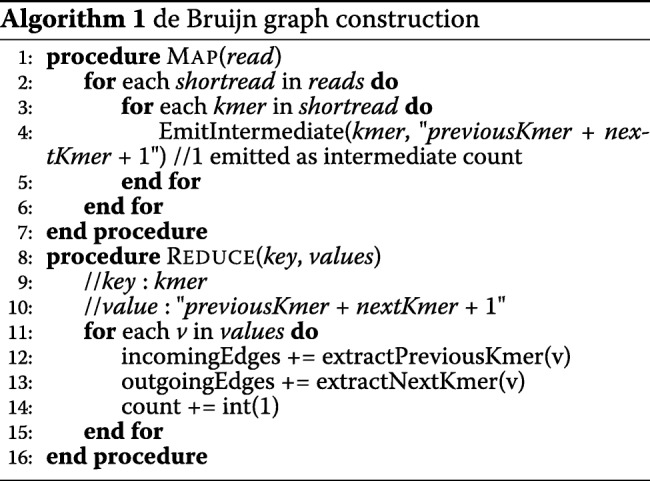


**Fig. 6:**
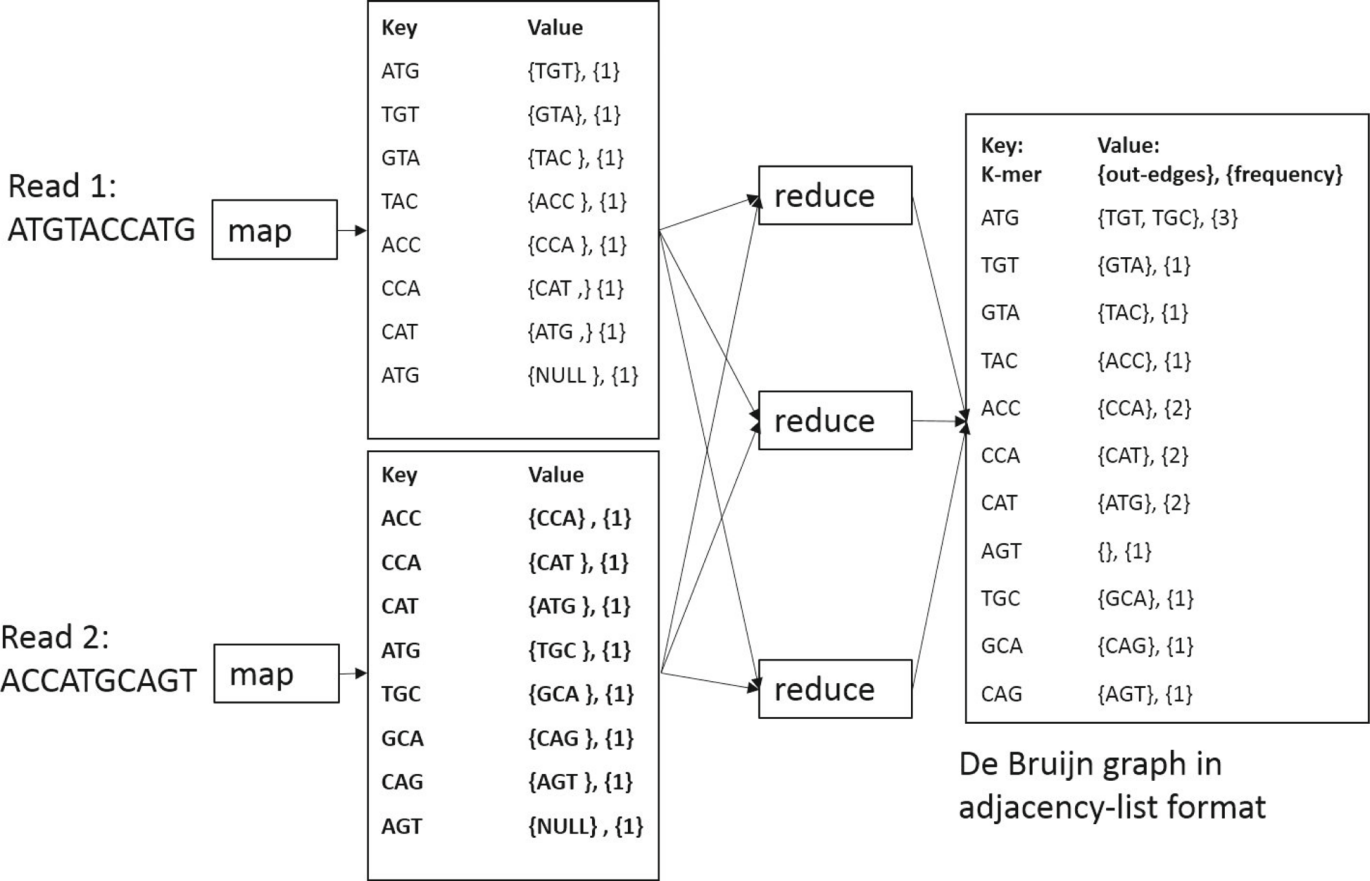
De Bruijn graph construction and *k*-mer count

Algorithm 1 explains the MapReduce algorithm for de Bruijn graph construction, and Fig. 6 shows the working of the algorithm. The map function scans each read of the data set and emits each *k*-mer as an intermediate key and its previous and next *k*-mer as the value. The intermediate key represents a vertex in the de Bruijn graph whereas the previous and the next *k*-mers in the intermediate value represent an incoming edge and an outgoing edge respectively. An associated count of occurrence (1) is also emitted as a part of the intermediate value. After the map function completes, the shuffle phase partitions these intermediate key-value pairs on the basis of the intermediate key (the *k*-mer). Finally, the reduce function accumulates all the previous *k*-mers and next *k*-mers corresponding to the key as the incoming and outgoing edges respectively. The same reduce function also sums together all the intermediate counts (i.e., 1) emitted for that particular *k*-mer. In the end of the reduce function, the entire graph structure and the count for each *k*-mer is stored in the NoSQL database of Hazelcast using Hazelcast’s *put* method. For improved performance, we emit only a single nucleotide character (i.e., *A*, *T*, *G*, or *C* instead of the entire *k*-mer) to store the incoming and outgoing edges. The actual *k*-mer can be obtained by prepending/appending that character with the *k*−1 prefix/suffix of the vertex *k*-mer.

### Locating the indel errors of long read

To locate the errors in the PacBio long reads, ParLECH uses the *k*-mer coverage information from the de Bruijn graph stored in Hazelcast. The entire process is designed in an embarrassingly parallel fashion and developed as a Hadoop Map-only job. Each of the map tasks scans through each of the PacBio reads and generates the *k*-mers with the same value of *k* as in the de Bruijn graph. Then, for each of those *k*-mers, we search the coverage in the graph. If the coverage falls below a predefined threshold, we mark it as weak indicating an indel error in the long read. It is possible to find more than one consecutive errors in a long read. In that case, we mark the entire region as weak. If the coverage is above the predefined threshold, we denote the region as strong or correct. To rectify the weak region, ParLECH uses the widest path algorithm described in the next subsection.

### Correcting the indel errors

Like locating the errors, our correction algorithm is also embarrassingly parallel and developed as a Hadoop Map-only job. Like LoRDEC, we use the pair of strong *k*-mers that enclose a weak region of a long read as the source and destination vertices in the DBG. Any path in the DBG between those two vertices denotes a sequence that can be assembled from the short reads. We implement the widest path algorithm for this local assembly. The widest path algorithm maximizes the minimum *k*-mer coverage of a path in the DBG. We use the widest path based on our assumption that the probability of having the *k*-mer with the minimum coverage is higher in a path generated from a read with sequencing errors than a path generated from a read without sequencing errors for the same region in a genome. In other words, even if there are some *k*-mers with high coverage in a path, it is highly likely that the path includes some *k*-mer with low coverage that will be an obstacle to being selected as the widest path, as illustrated in Fig. 1.

Therefore, ParLECH is equipped with the widest path technique to find a more accurate sequence to correct the weak region in the long read. Algorithm 2 shows our widest path algorithm implemented in ParLECH, a slight modification of the Dijkstra’s shortest path algorithm using a priority queue that leads to the time complexity of *O*(*E* log*V*). Instead of computing the shortest paths, ParLECH traverses the graph and updates the width of each path from the source vertex as the minimum width of any edge on the path (line 15).

### Locating the substitution error


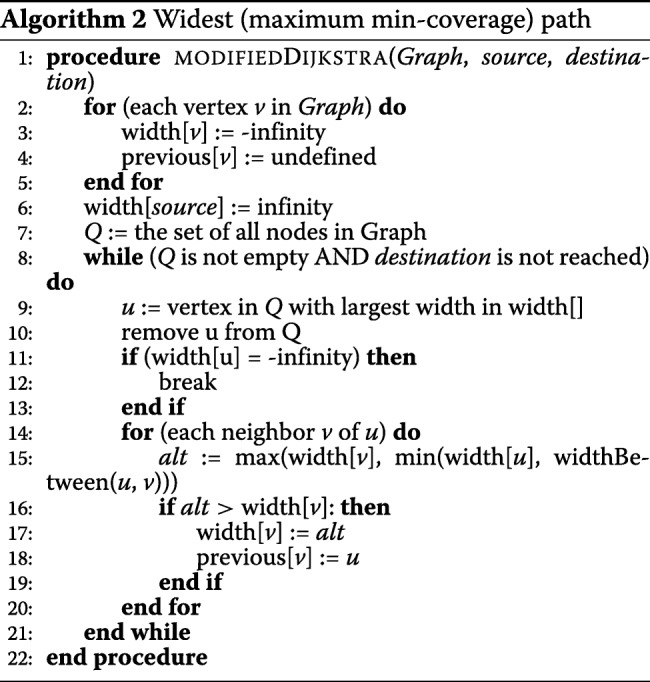



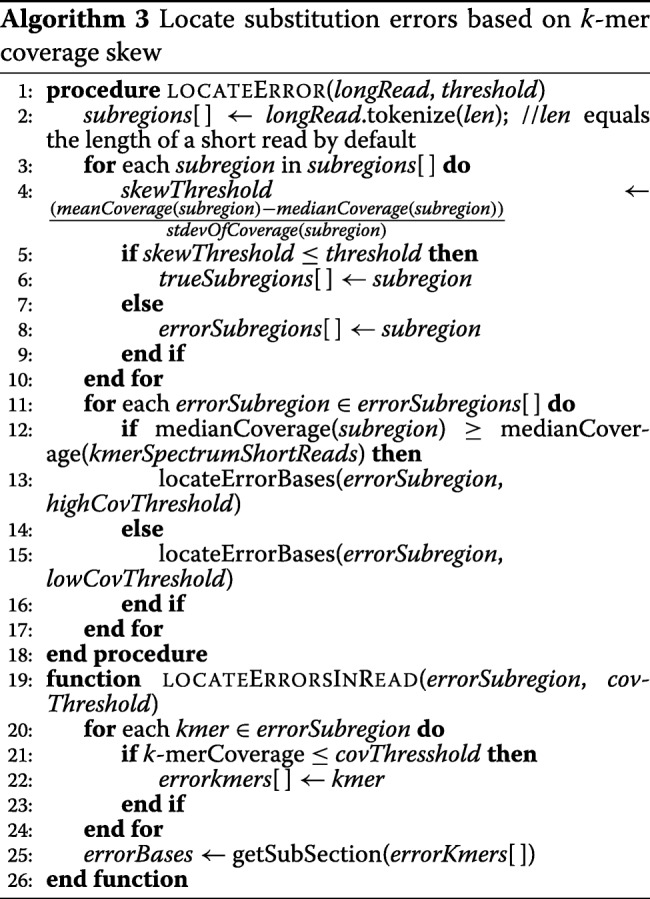


Algorithm 3 shows the process to locate substitution base errors. To locate the substitution errors in the long reads, we first divided the long reads into shorter fragments. As the *k*-mers in a smaller subregion tend to have similar abundances [27], this will divide the longer reads into a sequence of high- and low-coverage fragments. If a fragment belongs to a low-coverage area of the genome, most of the k-mers in that fragment are expected to have low coverage. Otherwise, the *k*-mers are expected to have high coverage. This methodology enables ParLECH to better distinguish between true-yet-low-coverage and error-yet-high-coverage *k*-mers. By default, ParLECH uses the length of the short reads as the length of the shorter fragments. However, it can be easily modified with a user-defined length. The last fragment of the long reads can have a length shorter than default (or user-defined) length. This fragment is always ignored for correcting the substitution error as it is considered insufficient to gather any statistics.

After dividing the long reads into shorter fragments, we calculate the Pearson’s skew coefficient (mentioned as *skewThreshold* in Algorithm 3) of the *k*-mer coverage of each fragment as a threshold to classify those fragments as true or error. If the skew coefficient of the fragment lies in a certain interval, the fragment is classified as a true fragment without any error. Furthermore, the fragments with mostly low-coverage *k*-mers are also ignored. All the other fragments (i.e., the fragments with highly skewed towards high-coverage *k*-mers) are classified as erroneous. Through this classification, all the low-coverage areas of the genome will be considered as correct even if they have low-coverage *k*-mers but almost similar coverage as that of the neighboring *k*-mers.

After classifying the fragments as true and error, we divide all the error fragments as high and low coverage. If the median *k*-mer coverage of a fragment is greater than the median coverage of the entire *k*-mer spectrum, the fragment is classified as high coverage. Otherwise, the fragment belongs to a low-coverage area. ParLECH uses a pattern of true and error k-mers to localize the errors and searches for the set of corrections with a maximum likelihood that make all k-mers true.

### Correcting the substitution error

To rectify the substitution errors, ParLECH uses a majority voting algorithm similar to that of Quake [4]. However, we have two major differences. First, ParLECH’s majority voting algorithm is fully distributed and can scale over hundreds of nodes. Second, unlike Quake, ParLECH uses different thresholds for the low and high coverage area of the genome to improve the accuracy. For each error base detected in the previous phase, ParLECH substitutes the base with all the different nucleotide characters (i.e., *A*, *T*, *G*, and *C*) and calculates the coverage of all the *k*-mers with that base. Finally, the error base is replaced with the one such that all those *k*-mers with that base exceeds or equals the specified threshold for that area.

## Results

In this section, we show the experimental results of ParLECH using various real-world sequence datasets.

### Datasets

We evaluate ParLECH with respect to four real data sets including *E. coli*, yeast, fruit fly, and human genome. The details of the data set are summarized in Table 1. The first three of them are relatively small-sized genomes. We use them to compare the accuracy of ParLECH with the existing hybrid error correction tools such as LoRDEC, Jabba, and Proovread. These data sets are also used to analyze the scalability and compare other resource consumption statistics such as memory requirement and CPU-Hour.

**Table 1:** Datasets

Data	Accn. #		#Reads		Data size (GB)		Read length		%Reads aligned	
	PacBio	Illumina	PacBio	Illumina	PacBio	Illumina	PacBio (Avg)	Illumina	PacBio	Illumina
E. coli	DevNet	ERR022075	282394	45440200	1.032	13.50	1120	101	78.97	99.44
Yeast	DevNet	SRR567755	2315594	4503422	0.53	1.20	5874	101	82.12	93.75
Fruit fly	BergmanLab	ERX645969	6701498	179363706	55	59	4328	101	51.14	95.56
Human	DevNet	SRX016231	23897260	1420689270	312	452	6587	101	72.3	79.60

The fourth one is the largest among all. It is a large human genome data set that consists of almost 764 GB of sequencing reads including both Illumina and PacBio sequences. We use it to showcase the scaling capability of ParLECH with hundreds of GBs of sequencing reads over hundreds of compute nodes. In our experiments, other existing tools could not produce the result for the data set.

### Computing environment

To evaluate ParLECH, we use *SuperMic* [30] HPC cluster, and Table 2 summarizes its configuration. The maximum number of compute nodes we can use for a single job is 128. Each node has 20 cores, 64 GB main memory, and one 250 GB hard disk drive (HDD). Note that the main bottleneck for our Hadoop jobs running on top of disk-based HDFS is the I/O throughput because each node is equipped with only one HDD. We expect that the performance of ParLECH can be significantly improved by using multiple HDDs per node and/or SSD. Our previous work [31–33] demonstrates the effects of various computing environments for large-scale data processing.

**Table 2:** Experimental environment

Maximum #nodes	128
Processor	Intel IvyBridge Xeon
#cores per node	20
DRAM per node	64 GB
Disk per node	250 GB hard disk drive
Network	56 Gbps InfiniBand

### Accuracy metrics

We evaluate the accuracy of ParLECH with respect to three different metrics as follows: 1) *% Aligned reads* and 2) *% Aligned bases*: These accuracy metrics indicate how well the corrected long reads are aligned to the reference genome. We report the %alignment both in terms of the total number of reads as well as the total bases present in the data set. For all the data sets other than the human genome, we use BLASR [34] to align the long reads to the reference genome as it reports longer alignments by bridging the long indel error. However, for the large human genome, we use BWA-mem [35] to get the alignment results quickly.

2) *N50 statistics:* It is also important to preserve input read depth in the corrected data set. Shorter reads and/or reduced depth may show better alignment but may have a negative impact on downstream analyses. Hence, we measure the N50 statistics of the data sets to indicate the discard or trimming of errors in the long reads instead of rectifying them.

3) *Gain:* We also use the **gain** metric [5] to measure the fraction of effectively corrected errors by ParLECH. The gain is defined as

1 $$ Gain = \frac{TP-FP}{TP+FN}  $$

where *TP* (true-positive) is the number of error bases that are successfully corrected, *FP* (false-positive) is the number of true bases that are wrongly changed, and *FN* (false-negative) is the number of error bases that are falsely detected as correct.

To measure *TP*, *FP*, and *FN*, we follow the procedure described in [36]. Let *r* be an original read and *r*_*c*_ be the read after correction. We derive the set of real sequencing errors *E*_*m*_ by mapping *r* to the reference genome and recording differences. Then, we measure *E*_*r*_, the set of errors remaining in *r*_*c*_, by applying global alignment between *r*_*c*_ and the genomic region where *r* was mapped to and recording the differences in the alignment. Finally, we calculate *TP*=|*E*_*m*_∖*E*_*r*_|, *FP*=|*E*_*r*_∖*E*_*m*_|, and *FN*=|*Er*∩*Em*|.

### Comparison with existing tools

Table 3 compares the accuracy of ParLECH with that of LoRDEC, Jabba, and Proovread in terms of the percentage of aligned reads and aligned bases. Table 4, on the other hand, compares the accuracy in terms of gain. We measure the accuracy metrics using BLASR by running multiple instances of BLASR in parallel for efficiently processing large datasets.

**Table 3:** Accuracy comparison (Alignments)

Data	Methodology	#Reads	#Bases	N50	#Aligned Reads	#Aligned bases	%Aligned reads	%Aligned bases
E. coli	Original	282394	316367409	3414	223017	237497013	78.97	75.07
	LoRDEC	282394	307987923	3422	247227	266373078	87.55	86.49
	Jabba	149836	149322524	2517	148293	141563938	98.97	94.80
	Proovread	263206	284871906	1222	241948	246138387	91.92	86.40
	ParLECH (Indel)	282394	309367145	3394	264574	285070391	93.69	92.15
	ParLECH (Indel+Subst)	282394	309367145	3394	264720	295438268	**93.74**	**95.50**
Yeast	Original	231594	1360457697	2990	190184	1206524663	82.12	88.69
	LoRDEC	231594	1345253694	2982	196669	1171490123	84.92	87.08
	Jabba	152882	634947441	2173	151359	634732955	99.02	99.09
	Proovread	225032	1307137185	1693	211323	1100350212	93.90	84.18
	ParLECH (Indel)	231594	1389446261	2994	199332	1240945939	86.07	89.31
	ParLECH (Indel+Subst)	231594	1389446261	2994	201857	1254987596	**87.16**	**90.32**
Fruit fly	Original	6701498	29007475325	15154	3427146	13355041639	51.14	46.04
	LoRDEC	6701498	30025673204	15154	3654326	14919815143	54.53	49.69
	Jabba	4423855	10820828565	14302	3921032	9455816742	88.63	87.38
	Proovread	6511617	20174923756	8603	5450784	14497076095	83.70	71.86
	ParLECH (Indel)	6701498	30117416348	15154	4417627	18799138439	65.92	62.42
	ParLECH (Indel+Subst)	6701498	30117416348	15154	4557627	19983756932	**68.01**	**66.35**

The best results are shown in bold faces

**Table 4:** Accuracy comparison (Gain)

		TP	FP	FN	%Gain
E. coli	LoRDEC	31264830	330659	4230385	87.15
	Jabba	10386868	105445	244608	96.7
	Proovread	23541209	318191	3942940	84.49
	ParLECH (Indel)	33229635	355464	3275190	90.05
	ParLECH (Indel+Subst)	34521649	250129	2088511	**93.61**
Yeast	LoRDEC	322660270	8989628	62594234	81.42
	Jabba	171200961	3004132	9543906	93.06
	Proovread	313517992	8734915	60820684	83.21
	ParLECH (Indel)	355708411	20037769	51642375	82.40
	ParLECH (Indel+Subst)	368206322	19556218	39626015	**85.49**
Fruit fly	LoRDEC	732799376	34190591	84891209	85.43
	Jabba	188817493	18141254	45042597	93.2
	Proovread	613007402	30867421	72123053	84.96
	ParLECH (Indel)	785735162	37126377	97826995	84.73
	ParLECH (Indel+Subst)	799834035	34065158	86789341	**86.37**

The best results are shown in bold faces

The results demonstrate that ParLECH can rectify the indel errors with significantly more accuracy comparing to LoRDEC both in terms of the aligned bases and gain. Like LoRDEC, ParLECH does not correct the long reads in which there is no strong *k*-mer. However, ParLECH searches strong *k*-mers in all reads regardless of their length while LoRDEC filters out reads whose length is less than a threshold.

Although Jabba attains significantly higher alignment accuracy compared to ParLECH, this high alignment accuracy is attained at the cost of producing reduced depths. This is because, unlike ParLECH, Jabba chooses to discard several of the uncorrected reads instead of rectifying them. As shown in Table 3, the total number of reads in the resulting error-corrected dataset is significantly higher in ParLECH comparing to Jabba.

Proovread attains almost similar alignment accuracy comparing to ParLECH. However, it trims many of the error regions in each read and breaks an erroneous longer read at the error region, producing multiple shorter reads. Consequently, Proovread produces significantly lower N50 compared to ParLECH.

We have further improved the accuracy by correcting the substitution errors of the long reads. This phase is not present in LoRDEC. However, it has a substantial impact on improving the quality of the data. As shown in Tables 3 and 4, by correcting the substitution errors, ParLECH improve the quality of the dataset by 1 to 3% from the indel error-corrected output both in terms of alignment and gain.

### Scalability

**Fig. 7:**
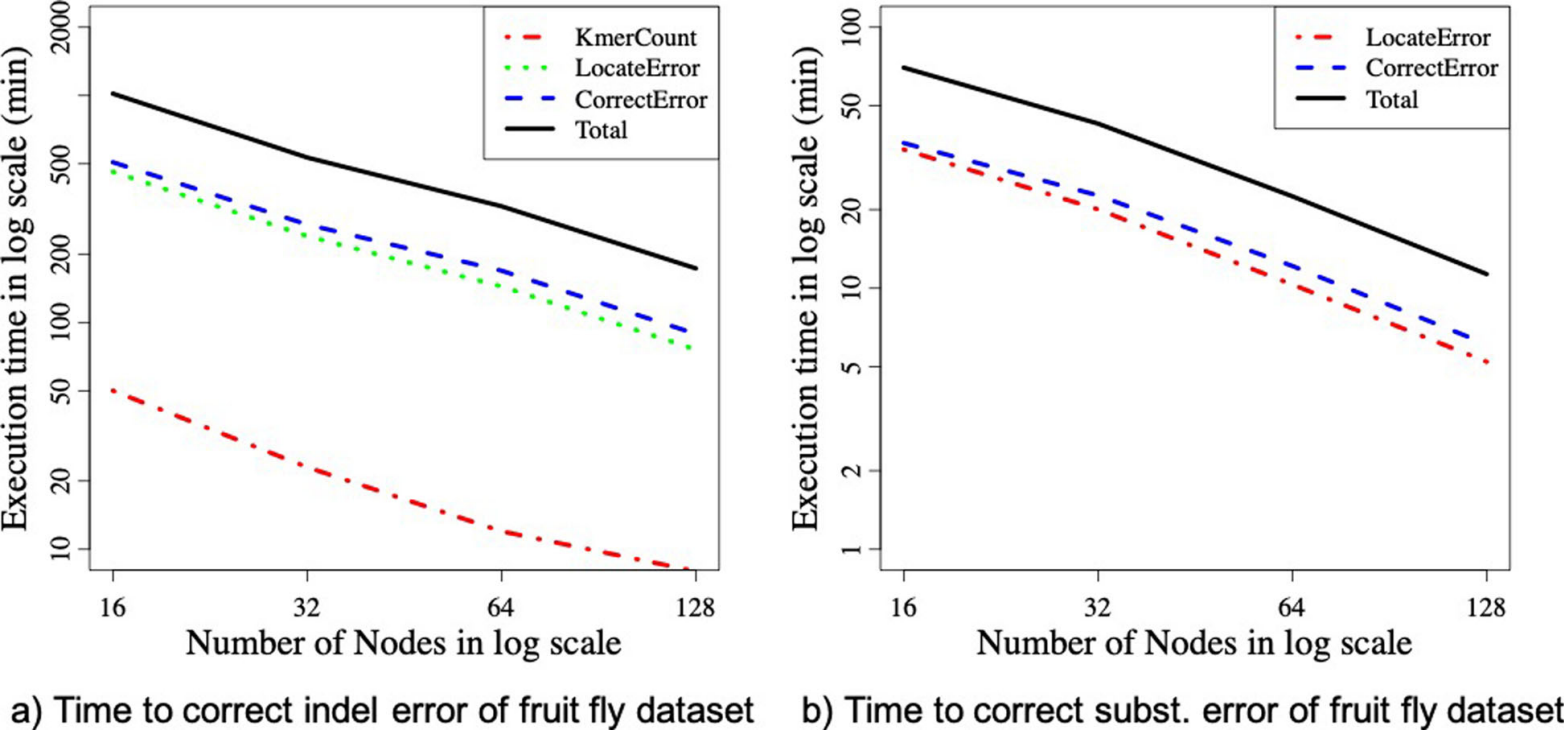
Scalability of ParLECH. **a** Time to correct indel error of fruit fly dataset. **b** Time to correct subst. error of fruit fly dataset

Figure 7 demonstrates the scalability of different phases of ParLECH. Figure 7a demonstrates the scalability of each phase of ParLECH’s indel error correction pipeline for the fruit fly dataset. The results show that the processing time of all three phases (i.e., constructing a de Bruijn graph, locating errors in long reads, and correcting errors in long reads) improves almost linearly with the increasing number of compute nodes. Therefore, the overall execution time of ParLECH also shows the almost linear scalability as we add more compute nodes.

Figure 7b demonstrates the scalability of different phases of ParLECH’s substitution error correction pipeline for the same fruit fly dataset. Like the indel error correction phases, these phases are also linearly scalable with the increasing number of nodes.

**Fig. 8:**
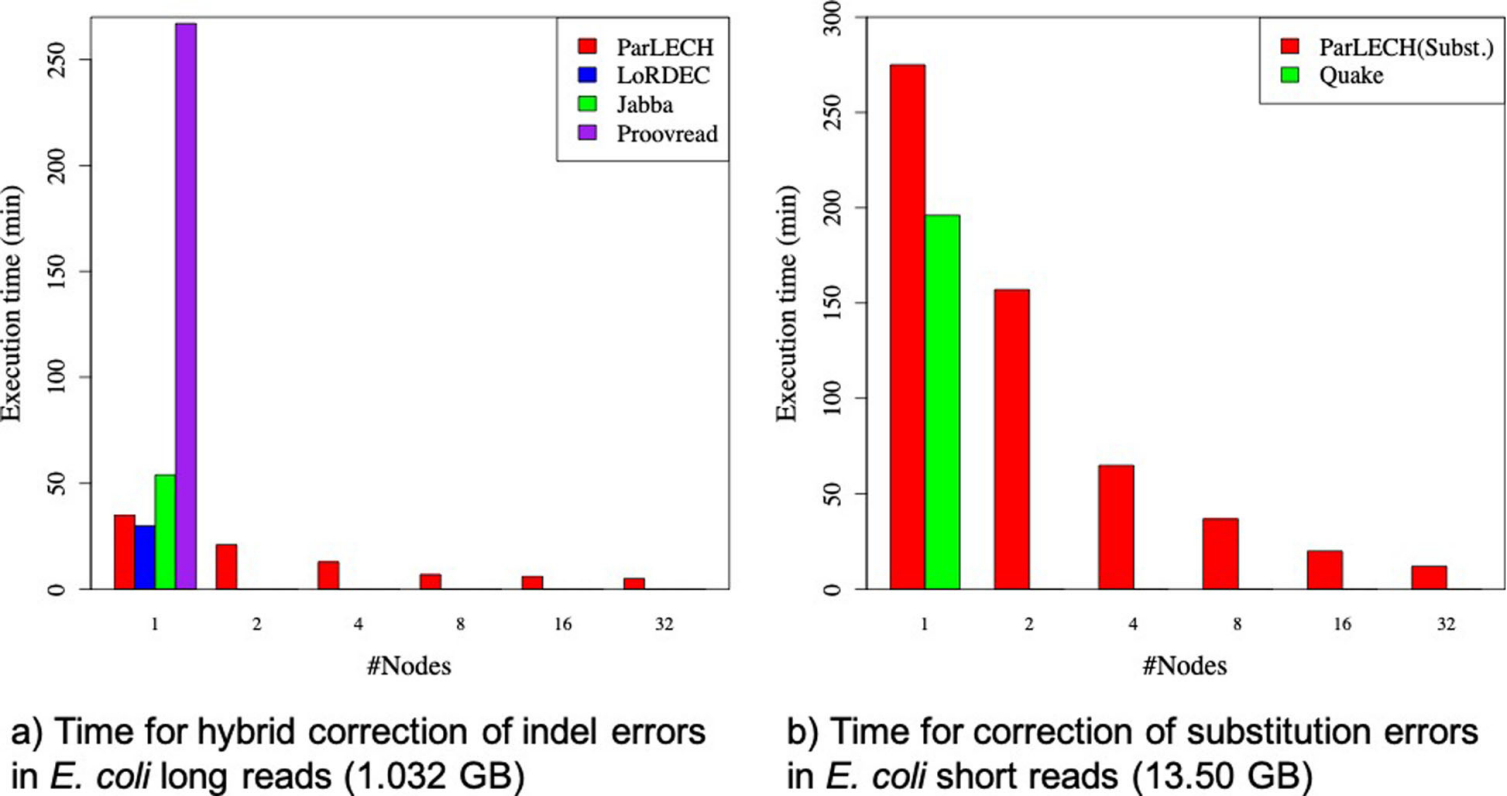
Comparing execution time of ParLECH with existing error correction tools. **a** Time for hybrid correction of indel errors in *E.coli* long reads (1.032 GB). **b** Time for correction of substitution errors in *E.coli* short reads (13.50 GB)

Figure 8 compares ParLECH with existing error correction tools. As shown in Fig. 8a, on a single node for the same *E. coli* data, ParLECH performs almost 1.5 times faster than Jabba and almost 7.5 times faster than Proovread. On a single node, LoRDEC shows slightly better (1.2 times faster) performance than ParLECH because both the tools have similar asymptotic complexity (*O*(*E* log*v*)) whereas ParLECH has some distributed computing overhead. However, utilizing the power of Hadoop and Hazelcast, the embarrassingly parallel algorithm of ParLECH can be easily distributed over multiple nodes and eventually outperform LoRDEC by several magnitudes, which is not designed for distributed computing. Even though the correction algorithm of LoRDEC can work independently on each of the long reads, the computation cannot be distributed because of the absence of a proper scheduler.

Figure 8b compares the substitution error correction pipeline with Quake [4], an existing tool to correct the substitution errors of Illumina short read sequences. For the similar reason mentioned above, ParLECH outperforms Quake by several magnitudes when distributed over multiple nodes. For a fair comparison with Quake, we use the *E. coli* Illumina dataset only for this experiment. Since the major motivation of ParLECH is to correct the long-read errors, we did not report the results of accuracy comparison between ParLECH and Quake in this paper.

## Discussion

### Effects of different traversal algorithms on indel error correction

To better understand the benefit of our widest path algorithm (ParLECH _*WP*_), we compare its accuracy with that of two other graph traversal algorithms, which are popular in this domain. The first one is the Dijkstra’s shortest path algorithm (ParLECH _*SP*_), and the other one is a greedy traversal algorithm (ParLECH _*Greedy*_). Table 5 reports the accuracy results of all the three algorithms over the real PacBio data sets.

**Table 5:** Effects of different traversal algorithms

Data	Methodology	#Reads	#Bases	#Aligned Reads	#Aligned bases	%Aligned reads	%Aligned bases
E. coli	ParLECH _*WP*_	282394	309367145	264574	285070391	93.69	92.15
	ParLECH _*SP*_	282394	307987923	247227	266373078	87.55	86.49
	ParLECH _*Greedy*_	282394	328966341	216543	233312807	76.68	70.92
Yeast	ParLECH _*WP*_	231594	1389446261	199332	1240945939	86.07	89.31
	ParLECH _*SP*_	231594	1355153783	196669	1171490123	84.92	86.44
	ParLECH _*Greedy*_	231594	1399628927	175478	1045262567	75.77	74.68
Fruit fly	ParLECH _*WP*_	6701498	30117416348	4417627	18799138439	65.92	62.42
	ParLECH _*SP*_	6701498	30193752318	3654326	14919815143	54.53	49.41
	ParLECH _*Greedy*_	6701498	32131749687	2946734	12030871508	43.97	37.44

ParLECH _*SP*_ replaces the weak region in the long read with the sequence corresponding to the shortest path in the DBG. ParLECH _*Greedy*_ always selects the vertex with the maximum coverage among all neighboring vertices during its traversal. For ParLECH _*Greedy*_, the traversal often ends up in a tip of a dead-end path. So, we use a branching factor *b* (100 by default) such that, after traversing *b* successive vertices from the source vertex, the algorithm backtracks if it cannot meet the destination vertex. The algorithm aborts when all successors from the source vertex are visited using this branching factor.

Although ParLECH _*SP*_ has the similar performance as ParLECH _*WP*_, because of the counter intuitive nature of shortest paths and the strong (high coverage) *k*-mers desired for the correction, it cannot take the advantage of the *k*-mer coverage information in a straight forward way, adversely impacting the accuracy. ParLECH _*Greedy*_, on the other hand, can take the advantage of the *k*-mer coverage information, but its accuracy depends highly on the higher value of the branching factor that poses a severe limitation on its performance.

Our widest path algorithm not only optimizes the performance but also makes better use of *k*-mer coverage information. The algorithm maximizes the minimum coverage of the *k*-mer in a path. Compared to both ParLECH _*SP*_ and ParLECH _*Greedy*_, ParLECH _*WP*_ better balances the coverage of all the *k*-mers in a particular path of the DBG, which improves the accuracy of the resultant data set.

As shown in Table 5, the widest path shows almost 15 to 25% better alignment accuracy compared to the greedy algorithm, which is found to perform worst among all. Comparing to the shortest path algorithm, the widest path shows almost 6 to 13% improvement for the dataset.

### Resource consumption statistics

Using the power of Hadoop and Hazelcast, ParLECH is capable to tradeoff between CPU-Hour and DRAM utilization. That is, based on the data size and the available resources, ParLECH can be tuned to utilize the disk space at the cost of higher execution time.

Table 6 compares the CPU-Hour and DRAM resource consumption of ParLECH with existing error correction tools with respect to the *E. coli* data set. For the best (lowest) execution time, ParLECH consumes almost similar CPU-Hour as LoRDEC, which is significantly less comparing to Jabba and Proovread. For this performance, ParLECH needs the entire *k*-mer spectrum in DRAM. Consequently, it utilizes almost 32GB of DRAM. However, ParLECH can process the same E. coli data consuming significantly less amount (only 5GB) of DRAM if configured properly. However, the process takes more time to finish because of context switching between the DRAM and the hard disk.

**Table 6:** Comparing resource consumption of ParLECH with existing error correction tools with respect to E. coli dataset

Error correction tool	CPU-Hour (single node)	Peak memory usage
LoRDEC	10	20.65
Jabba	18	11.16
Proovread	89	31.77
ParLECH (configured for least execution time)	11.67	23.80
ParLECH (configured to use lower DRAM)	29.37	5

### Processing large-scale human genomes

To showcase the data handling capability of ParLECH with hundreds of GBs of sequencing data and its scaling capability with hundreds of computing nodes, we analyze a large human genome data set. This 312 GB of PacBio data set includes more than 23 million long reads with the average length of 6,587 base pairs. The corresponding Illumina data set is 452 GB in size and contains more than 1.4 billion reads with the read length of 101 base pairs. To analyze this large data set (764 GB cumulative), we use 128 nodes of SuperMic cluster. We tuned ParLECH for the maximum performance. That means we distributed the entire de Bruijn graph in the memory available across the cluster.

The indel error correction process takes about 28.6 h as shown in Table 7. After this indel error correction, 78.3% of reads and 75.4% of bases are successfully aligned to the reference genome. The substitution error correction process took another 26.5 h, successfully aligning 79.73% of the reads and 80.24% of the bases to the reference genome.

**Table 7:** Correcting a human genome

PacBio data size	312GB
Illumina data size	452GB
#nodes used	128
Time	28.6 h
%Aligned reads (Indel)	78.3
%Aligned bases (Indel)	75.43
%Gain (Indel)	82.38
Time (Indel + Subst)	3.4 h
%Aligned reads (Indel + Subst)	79.73
%Aligned bases (Indel + Subst)	80.24
%Gain (Indel + Subst)	84.51

## Conclusion

In this paper, we present a distributed hybrid error correction framework for PacBio long reads, called ParLECH. For efficient and scalable analysis of large-scale sequence data, ParLECH makes use of Hadoop and Hazelcast. ParLECH uses the de Bruijn graph and *k*-mer coverage information from the short reads to rectify the errors of the long reads. We develop a distributed version of the widest path algorithm to maximize the minimum *k*-mer coverage in a path of the de Bruijn graph constructed from the Illumina short reads. We replace the indel error regions in a long read with their corresponding widest path. To improve the substitution accuracy, we develop a median statistics-based strategy that considers relative *k*-mer abundance in a specific area of a genome to take care of high- and low-coverage areas separately. Our experimental results show that ParLECH can scale with hundreds of compute nodes and can improve the quality of large-scale sequencing data sets in an accurate manner. While correcting the errors, ParLECH takes care of high- and low-coverage regions of the sequencing reads separately and is better capable to balance the *k*-mer coverage based on the neighborhood. Hence, we believe that it is a good starting point for detecting and correcting errors in RNA and metagenome sequences.

## Supplementary information

**Additional file 1** This file provides a brief of the theoretical rationale for using widest path algorithm (claim 1), and a theoretical justification for why median statistics has lower dependency on the value of *k*.

## Data Availability

The source code for ParLECH is available at https://github.com/arghyakusumdas/GenomicErrorCorrection.

## Abbreviations

CCT: Center for computation and technology
DBG: De bruijn graph
DNA: Deoxyribonucleic acid
DRAM: Dynamic random access memory
GB: Giga bytes
HDD: Hard disk drive
HDFS: Hadoop distributed file system
HPC: High performance computing
LSU: Louisiana State University
NoSQL: Not only SQL
ParLECH: Parallel long-read error correction using hybrid methodology
RNA: Ribonucleic acid
SSD: Solid state drive
UW: University of Wisconsin
