# Supplementary material for: A hybrid and scalable error correction algorithm for indel and substitution errors of long reads
Source: BMC Genomics. 2019 Dec 20;20(Suppl 11):948. doi: 10.1186/s12864-019-6286-9 (PMC6923905; doi:10.1186/s12864-019-6286-9)
Supplement: Supplementary file 1 — Additional file 1 This file provides a brief of the theoretical rationale for using widest path algorithm (claim 1), and a theoretical justification for why median statistics has lower dependency on the value of k. [file 12864_2019_6286_MOESM1_ESM.pdf]

RESEARCH

Additional File for Hybrid Correction of InDel and Substitution Errors of Long Read

Arghya Kusum Das*, Sayan Goswami, Kisung Lee and Seung-Jong Park

*Correspondence:
dasa@uwplatt.edu
Full list of author information is available at the end of the article
†Equal contributor

Appendix

0.1 A brief theoretical rationale of Claim 1 (use of widest path):

There are k k-mers of R_1 between pos_{begin} and pos_{end}, and we can assume that their occurrences (i.e., coverage) are independent and identically distributed (i.i.d.) random variables expressed as x_1, x_2, x_3, x_4, etc. Hence, the minimum of all the occurrences of those k k-mers can be expressed as

$$Y = min(x_1, x_2, x_3, ..., x_n). \tag{1}$$

The corresponding distribution of Y i.e., the minimum distribution of the k-mers is

$$P(Y \leq x) = 1 - (1 - F(x))^n \tag{2}$$

where $F(x)$ denotes the distribution of the coverage of the k k-mers.
Assuming, $F(x)$ follows an exponential distribution with rate r i.e. $F(x) = 1 - \exp(-rx)$,

$$P(Y \leq x) = 1 - \exp(-\mu x) \tag{3}$$

Where, $\mu = rk$.
For a carefully chosen cut off point (as discussed in [1] and [2]) where the ratio of error k-mers to true k-mers is high, the value or r at $R1$ is significantly higher than that in $R2$. That means, the probability of having the k-mer with minimum coverage is higher in R_1 comparing to R_2 which justifies the claim. Although we consider the reads represent the same region in the genome for the sake of convenience and easy understanding, the claim can be easily justified for any reads with overlap where one of the reads has the error base in the overlapped region.

0.2 A brief theoretical justification for why median-based substitution-error detection has lower dependency on k-mer size

The total number of k-mers generated from a read of length l

$$n_k = l - k + 1 \tag{4}$$

After sorting all the k-mers of the read in ascending order of their occurrences (i.e., coverage) in the entire short read dataset (obtained during k-mer counting), the position of the median can be expressed as,

$$p_{med} = \frac{n_k}{2} \tag{5}$$

The left side of p_{med} presents the k-mers with lower abundance. All the k-mers with errors will fall in this left side. Let us consider, for a maximum of e_{max} base errors the median statistics will remain unaltered. Hence, we can say,

$$k.e_{max} \leq p_{med} \tag{6}$$

where each error base may correspond to maximum k k-mers.

$$\implies e_{max} \leq \frac{p_{med}}{k} \tag{7}$$

It means, in case of a minimum of 2bp errors in an Illumina read of 100bp (which is common), k can be chosen any number between 15 and 19. However, it should not be very small. In average, we got good and almost invariant accuracy for most of the genome sequences with k greater than 10.

Author details
[1]Department of Computer Science and Software Engineering, University of Wisconsin at Platteville, USA. [2]School of Electrical Engineering and Computer Science, Center for Computation and Technology, Louisiana State University, Baton Rouge, USA.

References

1. Chin, F.Y., Leung, H.C., Li, W.-L., Yiu, S.-M.: Finding optimal threshold for correction error reads in dna assembling. BMC bioinformatics **10**(1), 15 (2009)
2. Kelley, D.R., Schatz, M.C., Salzberg, S.L.: Quake: quality-aware detection and correction of sequencing errors. Genome biology (2010)
